# Effects of a secondary mental task and additional auditory feedback on body movements and EEG

**DOI:** 10.1007/s00221-026-07313-x

**Published:** 2026-05-04

**Authors:** Swapno Shadman Aditya, Adam Clarke, Lucy Armitage, Evangelos Pappas, Victoria Traynor, Winson Chiu-Chun Lee

**Affiliations:** 1 School of Engineering, University of Wollongong, Wollongong, 2522 Australia; 2 Advanced Mechatronics and Biomedical Engineering Research Group, University of Wollongong, Wollongong, 2522 Australia; 3 School of Psychology, University of Wollongong, Wollongong, 2522 Australia; 4 Graduate School of Biomedical Engineering, University of New South Wales, Sydney, NSW 2033 Australia; 5 School of Health and Biomedical Sciences, RMIT University, Melbourne, 3001 Australia; 6 Faculty of Medicine and Health, The University of Sydney, Sydney, Australia; 7 Shanghai University of Medicine and Health Sciences, Shanghai, China; 8 University of the Sunshine Coast, Sunshine Coast, 4560 Australia; 9 Warrigal, Illawarra, Albion Park Rail, 2527 Australia

**Keywords:** Dual task, Biofeedback, Electroencephalogram, Movement analysis

## Abstract

Previous studies have shown that additional cognitive load from a secondary task can adversely affect movement performance. However, how externally provided auditory pacing influences motor and neural responses under dual-task conditions remains unclear. This study employed a repeated-measures experimental design, studying eighteen young adults (Mean age 23.5 ± 4 years) who underwent three conditions: (1) foot tapping only (single task), (2) foot tapping and a mental task (dual task), and (3) foot tapping, mental task, and auditory pacing biofeedback (dual task + biofeedback). Ankle joint movements using Xsens IMU’s (Inertial Motion Units) and brain activities using EEG (electroencephalography) were measured in these three conditions. Results showed that dual tasks significantly reduced (*p* < 0.01) the range of motion and increased (*p* < 0.05) the variability of ankle joint range of motion, suggesting a decline in foot tapping performance compared to the single-task condition. The decline was accompanied by significant increases (*p* < 0.05) in relative high-beta power in EEG, consistent with heightened cognitive-motor demand during dual-tasking. In the dual-task + biofeedback condition, kinematic measures returned to values statistically indistinguishable from the single-task condition and response times in the cognitive task were significantly reduced, without a loss of accuracy. The relative high-beta power was also significantly reduced, compared with the dual-task condition, which may reflect increased entrainment to external cues or a reduction in cognitive load. These results support the role of auditory pacing in facilitating movement performance under dual-tasking conditions, while highlighting the need for future studies to dissociate entrainment effects from changes in cognitive workload.

## Introduction

Multitasking refers to performing more than one task simultaneously, switching between tasks, or a combination of both (Carrier et al. 2015). The simple act of driving, where a person must not only maintain ancillary movements and controls with their feet but also scan and concentrate on the road ahead, is an example of multitasking. While performing a single task/standalone task, the brain employs multiple networks, including the frontoparietal control network, the dorsal attention network, and the ventral attention network, to handle different aspects such as planning, focusing on sensory information, and attention reorientation (Leone et al. 2017). However, in the case of dual tasking or multitasking where more than one task is involved, there is an effect of ‘bottleneck theory’, which states that the human brain undergoes both structural and functional limitations during the handling or processing of multiple information at once (Dux et al. 2009; Szameitat et al. 2016). As a result, a reduced performance in cognitive or motor task (Or both) can be observed (Boyer et al. 2023; Hung et al. 2013). This occurs due to the forementioned networks getting stressed due to the ‘crosstalk’ interference effect which stems from dual tasking or multitasking, or more essentially, processing multiple information simultaneously (Leone et al. 2017).

Previous studies have investigated how dual-tasking (performing two different tasks concurrently) can affect walking and lower limb movement performance (Kimura and Duersen 2020; Nordin et al. 2010). While walking, a distraction from a secondary task, such as using a phone, caused negative changes in lower limb movements and walking among both young (Wang et al. 2022; Ruffieux et al. 2015) and elderly people (Beurskens and Bock 2012; Springer et al. 2006), which can lead to falls (Lundin-Olsson et al. 1998; Weerdestyne et al. 2003). Meanwhile, driving requires attentional resources to be allocated to visual, cognitive, and action processing (Kimura et al. 2022). A review conducted by Engstrom et al. (2017) found many studies that associated increased cognitive load with driving mishaps, such as improper lane changes.

Other studies have measured how dual tasks affected responses in simpler tasks. It was found that an additional color identification task significantly increased foot off and foot contact time during voluntary step execution while standing on a force plate (Melzer et al. 2007). While performing foot stepping in a seated position, an additional cognitive task involving mathematical operations reduced movement accuracy and increased brain activation (Ohsugi et al. 2013). Furthermore, the ability to perform rhythmic foot taps precisely was suggested as an accurate measure of cognitive load during concurrent mental tasks (Park & Brunken 2014).

There are devices providing users with biofeedback, which remind users of their errors in body movements, and have been shown to have positive effects on gait (Giggins et al. 2013). For example, when providing feedback on the degree of postural sway, gait parameters, including velocity and cadence, were found to improve (Verehoeff et al. 2009; Lee et al. 2015). Meanwhile, some biofeedback devices rely solely on rhythmic auditory cues that provide temporal guidance without conveying explicit movement errors, and they have also been shown to improve gait symmetry (Gouda and Andrysek 2024). Biofeedback was also found to improve driving. In particular, Wang et al. (2019) found that auditory feedback improved drivers’ response time and visual feedback improved driving accuracy.

Different theories explain the benefits of biofeedback or cue-based interventions. Certain forms of biofeedback decrease cognitive load (Fuentes-García et al. 2025), and decreased cognitive load actually links to improved performance (Eesee et al. 2025). Meanwhile, some other studies suggest that rhythmic auditory cues may function not simply as reduced cognitive load, but as external temporal constraints that support performance through sensorimotor entrainment, and adaptive reallocation of cognitive–motor resources. These are consistent with recent work demonstrating EEG modulation during auditory cued movement (Tharawadeepimuk et al. 2024), adaptive cognitive–motor resource reallocation during dual task walking (Patelaki et al. 2022), and improved timing stability through auditory pacing in dual task tapping (Mudarris et al. 2025). There is also a literature review highlighting cueing and entrainment frameworks (Scataglini et al. 2025).

While biofeedback has been shown to improve motor performance and auditory pacing to modulate neural activity, it remains unclear how these effects manifest when the brain is already loaded with dual task demands, and whether neural changes observed under such conditions correspond to improvements in motor performance. The main aims of our study was to investigate the effects of dual-task and dual-task + biofeedback on lower-limb movements (foot tapping) and brain activities. While it is expected that dual-tasking impairs lower-limb movements and increases brain electroencephalography (EEG) activities, this study focuses on exploring whether lower-limb movements and EEG activities change and improve during the dual-tasking + biofeedback condition.

Foot tapping was chosen as the lower-limb movement in this study, as it has been shown to have good specificity in identifying changes in cognitive ability and load (Mancioppi et al. 2022). Furthermore, foot tapping involves inhibitory processes, which can be good indicators of cognitive processing control (Cohen et al. 1997) and thus can help us understand cognitive load during a dual-task scenario involving a secondary task. This article aims to describe experimental protocols, detailing different conditions to be tested involving single (foot tapping) and dual tasks, as well as dual tasks aided by biofeedback. It then reports and discusses the biomechanical results regarding the ankle joint movements, along with EEG results.

## Methods

### Participants

Eighteen healthy young adults (Mean age 23.5 ± 4 years; 13 females and 5 males) participated in the experiment. A post hoc power analysis was conducted using G Power software (version 3.1.1). Assuming a repeated measure MANOVA design with one group and 3 separate conditions, a medium effect size of 0.4 and a significance level of 0.05, the resulting statistical power was 0.75 for a sample size of 18 participants. Effect size was chosen due to its effectiveness in terms of clinical significance (Sullivan and Feinn 2012; Lininger and Riemann 2016). All participants scored above 24 (mean score = 28) out of 30 in the Standard Mini-Mental State Exam (SMMSE), suggesting they were cognitively healthy. All participants had no prior history of any traumatic head injuries or any recent lower limb injuries. They did not participate in any exercise or physical activity that intensively involved the leg muscles, at least 24 h before participating in the experiment. 13 out of 18 participants participated in the study as part of the University of Wollongong Psychology department’s extra credit program, and five other participants were obtained through expression of interest in participating from the community. Ethics approval was obtained from the University of Wollongong’s HREC (Human Research Ethics Committee, Ref no. 112/2023).

### Experiment procedure

A repeated measures experimental design was used such that each participant experienced all 3 conditions on the same day: (1) foot tapping (single task), (2) foot tapping (dual task), and (3) foot tapping (dual task + biofeedback). The order of these tasks was randomized for each participant. Between tasks, there was a 2-minute rest interval.

#### Single-task condition (ST)

The participants sat in a chair, feet resting on the floor, which was selected for comfortable sitting. Foot tapping was performed by first tapping the toes of both feet twice simultaneously, while their heels rested on the floor. They then tapped their heels twice while their toes rested on the floor. This tapping pattern was chosen as it involves the participant concentrating on the moment they have to switch tapping from their heels to their toes, introducing extra cognitive load. The participants were instructed to tap at a natural and comfortable pace. This is because in the case of gait analysis studies, natural walking pace has proven to be an effective baseline compared to externally influenced walking pace, in terms of physiological aspects (Majed et al. 2024; Graham et al. 2008).

The foot tapping was conducted for two minutes. The number of taps was counted to determine the frequency of the foot taps.

#### Dual-task condition (DT)

In the Dual Task (DT) condition, participants performed the same foot-tapping task as in the single-task condition while carrying out a stimulus-based flanker task as a secondary task. In the arrow flanker cognitive task, five arrows pointing either left or right were presented in a row on a computer screen (Eriksen and Eriksen 1974). The participants were asked to focus on the middle arrow and, as quickly as possible, indicate whether it pointed left or right using a handheld controller. The arrows were presented for 1.5 s, then another set of arrows appeared for the participants to respond. An interval of 1 s was present between each arrow stimulus. The timing of the stimulus and the participant’s response (Pressing the buttons) were recorded in the EEG data as event markers. The entire task lasted two minutes.

#### Dual task + biofeedback condition (DT+)

In the dual task + biofeedback (DT+) condition, participants performed the same tasks as in the dual task condition, while being instructed to do their best to match each tap to the auditory feedback. The auditory feedback was provided using a metronome. The metronome frequency for the DT+ condition was determined by a one-minute video-recorded foot-tapping session, performed before each participant participated in the three tested conditions. During the session, the participants performed foot tapping at their self-selected comfortable frequency. A tap was defined as one complete foot-to-ground contact. The total number of taps was counted by visual inspection of the video and divided by the recording duration (60s) to obtain the tapping frequency. Throughout the entire DT+ condition, the same metronome frequency was used for each individual participant. The DT+ condition provided baseline-calibrated auditory pacing, hereafter referred to as auditory pacing biofeedback. The auditory cue did not reflect movement error or physiological state.

### Experimental equipment and measurements

#### Kinematic data collection (XSens)

**Fig. 1:**
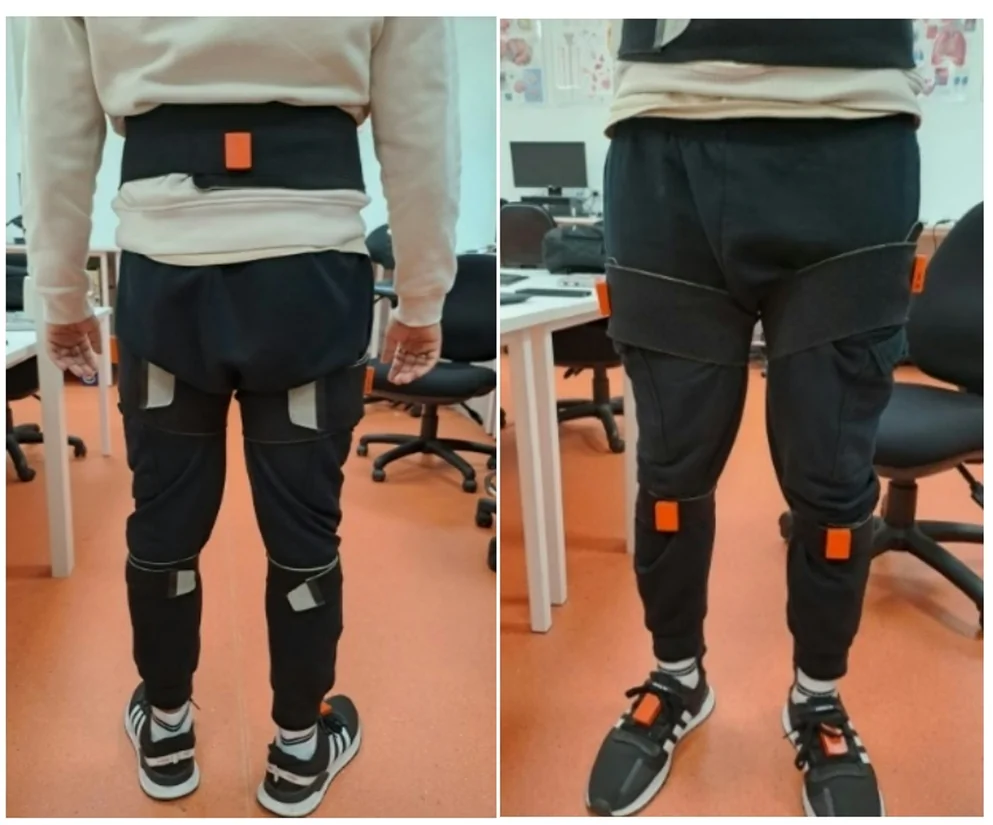
Participant wearing XSens sensors on lower limb

XSens motion-tracking sensors were attached to the participants’ pelvis, knee, and ankle joints to track foot-tapping movements at a sampling rate of 100 Hz. Seven lower limb sensors were used. One sensor was attached to the back of the pelvis, and the other six sensors were attached across both legs (One sensor on the upper thigh, one sensor just below the knee joint, and one sensor attached to the ankle). Their locations are shown in Fig. 1.

#### EEG setup

**Fig. 2:**
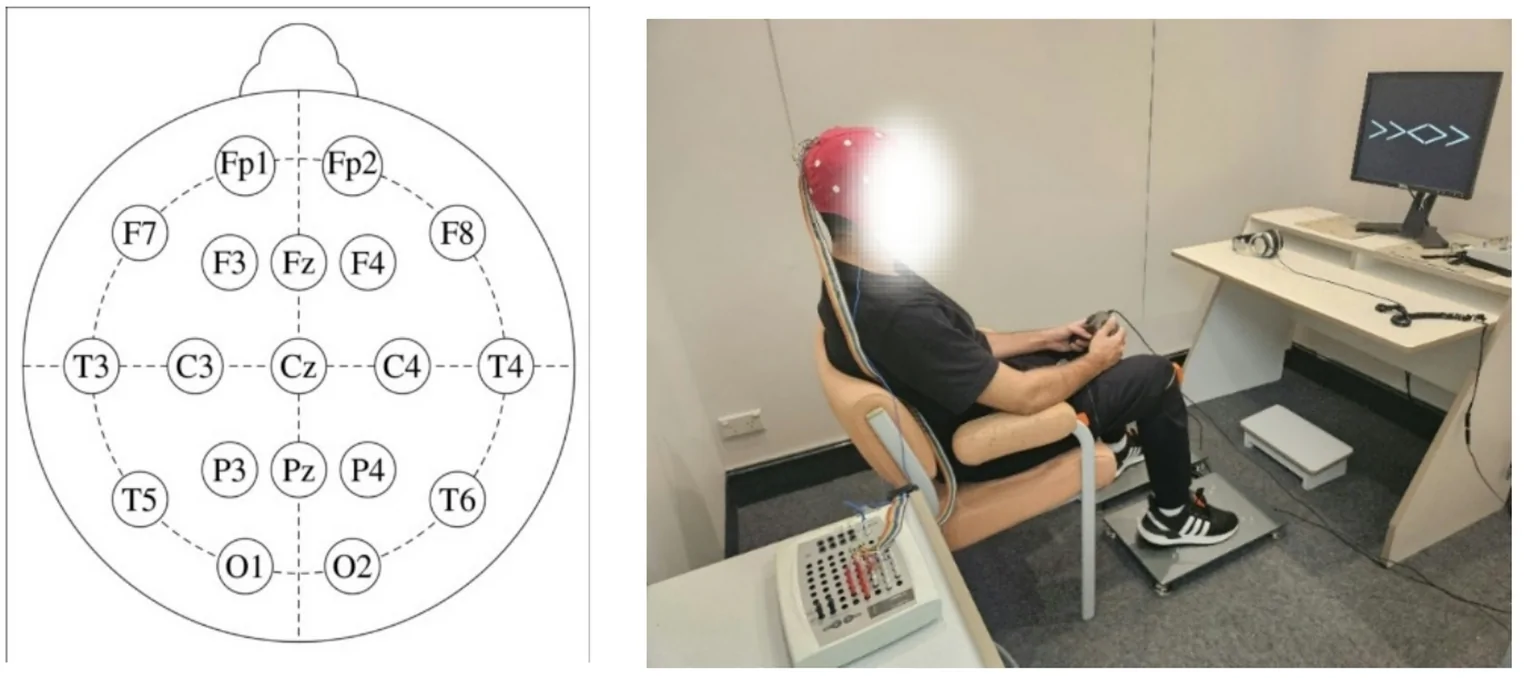
19 channel 10/20 EEG electrode distribution (left) (tdcs.com, 2025), participant performing the flanker task and foot tapping with EEG cap and XSens sensors on (right)

EEG Recordings were conducted using a Neuroscan’s SynAmps2 amplifier and Acquire software (version 4.5.1). The EEG was recorded from DC to 70 Hz, with a notch filter at 50 Hz, and sampled at 1000 Hz. EEGs were recorded from 19 channels in accordance with the International 10/20 system using tin disc electrodes (Fig. 2). All electrodes were referenced to A1, and A2 was recorded as the active electrode. A ground electrode was located between Fpz and Fz. Four electrodes were fitted above and below the right eye, and on the outer canthi of both eyes to record activity. Eye movement EEG data, which consisted of vertical and horizontal eye movement as well as eye blinks, were recorded separately from the experimental EEG data for use as a reference for calibration. 

### Data processing and analysis

#### Ankle motion data from Xsens

Data from XSens MVN 2019 were filtered by a second-order Butterworth filter with a cutoff frequency of 5 Hz. This considered suggestions from Winter (2009) that second-order Butterworth low-pass filters were accurate for processing biomechanical signals. Furthermore, cut-off frequencies around 5 Hz have been utilized in the literature (Erer 2007; Tong and Granat 1999; Crenna et al. 2021) and in movement analysis software such as OpenSim (Delp et al. 2007). Within 120 s of foot tapping, movement data between 40 and 120 s were analyzed. This was done because, at the beginning of the trial, drifts were observed in the EEG and IMU signals, and participants needed time to get comfortable with their tapping. Our observations also indicate slightly better foot-tapping performance among participants after an initial 40-second period.

Further processing of the data was conducted in MATLAB 2023b to compute (1) the mean ankle range of motion (ROM), (2) the mean angular velocity, and (3) the coefficient of variation (COV) of ankle ROM and velocity. The range of motion and angular velocity were output directly from the Xsens *MVN2019* software, which defined the ankle ROM of each tap as the difference between the peak dorsiflexion and plantarflexion angles and the angular velocity as the division of the joint ROM by the duration of that step. The mean values were obtained by first taking the average across all steps and then across all 18 participants. COV of both ankle joint ROM and angular velocity was calculated for each participant by dividing the standard deviation by the mean value of the data, and the average COV values across the 18 participants for each parameter were reported.

#### EEG data

The EEG data for each participant and each condition were initially processed using Neuroscan 4.5 software. The RAAA algorithm (Croft and Barry 2000) was used to correct eye-movement artifacts from the data. The EEG data were then digitally re-referenced to the linked ears, and any bad channels which showed high levels of muscle artifact (e.g., T3,T4 channels showing neck and facial muscle artifacts) were interpolated using linear derivation in the Neuroscan 4.5 software, which replaced the bad channels’ data with the average of the two nearest channels. Following the eye-movement correction, an experienced technician visually appraised the EEG to determine whether any artifact remained. The processed EEG data were then Fourier transformed using Neuroguide version 2.6.1. From the corrected EEG, a minimum of 60 s of artifact-free EEG was selected for analysis, from which 2s epochs were Fourier transformed using a Hamming window. The first 40 s of the recording were not analyzed, staying consistent with Xsens signal processing timings. However, after this period epochs were selected from across the entire trace to prevent the possibility of timing effects being inadvertently added to the analysis. The system extracted the EEG spectral power for the Beta (13–24 Hz) and High Beta (24–39 Hz) frequency bands for relative power. Beta oscillations play a central role in cognitive–motor integration and attentional control during movement, while relative power can reflect oscillatory behavior of the brain, and does take into account external error sources such as skull or hair thickness (Sandre and Troller-Renfree 2025).

#### Statistical analysis

Multivariate analysis of variance (MANOVA) using IBM SPSS Statistics (Version 28.0.0.0) was conducted to identify if there were significant differences across the 3 test conditions (single-task, dual-task, and dual-task + biofeedback conditions) for both kinematic and EEG parameters. Normality was assessed using the Shapiro-Wilk test supplemented by visual inspection of Q-Q plots applied to the model residuals. All residual distributions satisfied the normality assumption (Shapiro-Wilk test, all *p* > 0.05), and visual inspection indicated only negligible deviation, supporting the use of MANOVA. Kinematic MANOVA included left and right ankle joint ROM, velocity and COV (8 parameters). EEG MANOVA included beta and high beta relative power averaged across global (all electrodes), central (C3, Cz and C4, Fz and Pz), frontal (FP1, FP2, F3, F4, F7, F8), and posterior (P3, P4, P7 and P8) brain regions (8 parameters). If MANOVA indicated a significant difference, ANOVA followed by pairwise comparisons with the Benjamini-Hochberg Procedure to control the False Discovery Rate (FDR) was performed. Each dependent variable was treated as a separate statistical family, and FDR correction was applied only to the three pairwise condition comparisons within that variable. The probability (*p*) values from pairwise t-tests were ranked (Rank) from lowest to highest, and significant differences were declared only if the *p* values were below the Benjamini-Hochberg critical values (Rank x Q /n), where Q was the false discovery rate set as 0.05, and n is the number of comparisons (3).

The use of a hierarchical testing framework, in which pairwise comparisons were only conducted when significant MANOVA and ANOVA results were present, served as a gatekeeping approach. This substantially reduced the multiplicity burden and justified limiting FDR control to the three condition-level comparisons within each significant dependent variable.

## Results

### Foot-tapping kinematic results

**Table 1:** Average ankle joint angle range of motion and angular velocity reported with margin of error with a 95% confidence interval, along with the standard deviation values (in brackets) across 18 participants

Parameter/values (mean ± SD)	Single task	Dual task	Dual task + biofeedback	*p* value
Left ankle joint ROM (degree)	12.04 ± 5.5	9.05 ± 5.7	11.09 ± 6.71	0.005*
Right ankle joint ROM (degree)	11.65 ± 5.35	9.3 ± 5.18	11.13 ± 5.30	0.002*
Left ankle joint velocity (rad/s)	0.064 ± 0.036	0.063 ± 0.043	0.059 ± 0.044	0.7
Right ankle joint velocity (rad/s)	0.061 ± 0.022	0.049 ± 0.014	0.058 ± 0.022	0.04*

Parameters with * indicate significant differences among the 3 conditions through ANOVA

Table 1 shows the kinematic values of ankle joint ROM and angular velocity in all 3 test conditions. The repeated-measures MANOVA revealed a significant effect of condition on ankle kinematics (F(8, 62) = 3.07, *p* = 0.006). Follow-up univariate ANOVA showed significant effects of Condition for both left ankle range of motion (F(2, 34) = 6.09, *p* = 0.006), and right ankle range of motion (F(2, 34) = 7.30, *p* = 0.002). Both left and right ankle ROM showed a significant decrease (*p* < 0.01) during the dual task condition, and a significant increase (*p* < 0.05) was observed during the auditory pacing biofeedback condition, reaching levels similar to those observed in the single task condition. Meanwhile, right ankle velocity was significantly affected by condition (F(2, 34) = 3.46, *p* = 0.043). Post-hoc pairwise comparisons with Benjamini–Hochberg correction indicated that right ankle velocity was significantly lower in the dual-task condition compared with the single-task condition (*p* < 0.01), whereas no significant difference was observed between the dual-task and dual-task + biofeedback conditions.

Similar results were observed in the coefficient of variation (CoV), as shown in Table 2. Significant differences were observed in right (F = 5.52, *p* = 0.008, η^2^*p* = 0.2453; Medium-Large effect) and left (F = 3.61, *p* = 0.037, η^2^*p* = 0.176; Medium-Large effect) ankle joint angle CoV, revealed by repeated measures ANOVAs. Post-hoc pairwise comparisons with Benjamini–Hochberg correction further revealed significantly larger CoV values (*p* < 0.05) in the dual-task condition, compared to both the single-task and dual task + biofeedback conditions for right ankle joint angle (DT > ST, DT+). No significant differences were observed for the left ankle joint CoV using FDR corrections, as well as the CoV for left and right ankle joint angular velocity.

**Table 2:** Average coefficient of variance of ankle joint angles and angular velocities

Parameter	Single task	Dual task	Dual task + biofeedback	*p* values
Left ankle joint angle CoV	0.56 ± 0.12	0.72 ± 0.33	0.59 ± 0.16	0.037*
Right ankle joint angle CoV	0.56 ± 0.15	0.64 ± 0.17	0.56 ± 0.12	0.008*
Left ankle joint velocity CoV	1.38 ± 0.53	1.25 ± 0.29	1.34 ± 0.62	0.5
Right ankle joint velocity CoV	1.16 ± 0.24	1.12 ± 0.18	1.155 ± 0.12	0.7

Mean values shown with margin of error for 95% confidence interval, and SD values in brackets. * indicates significance measured in ANOVA

### Flanker task performance

**Fig. 3:**
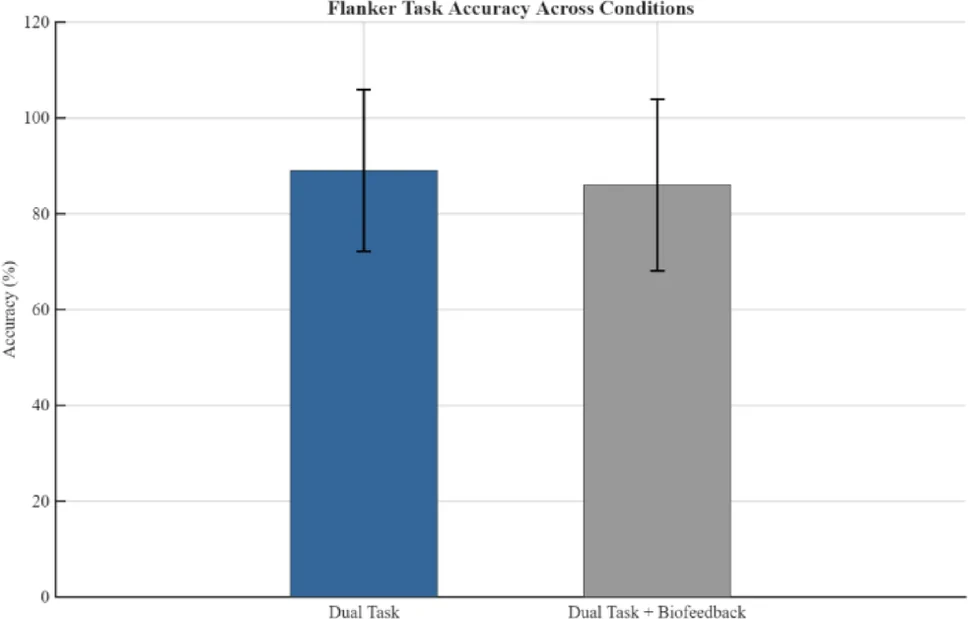
Flanker task performance for dual task and dual task + biofeedback condition

**Fig. 4:**
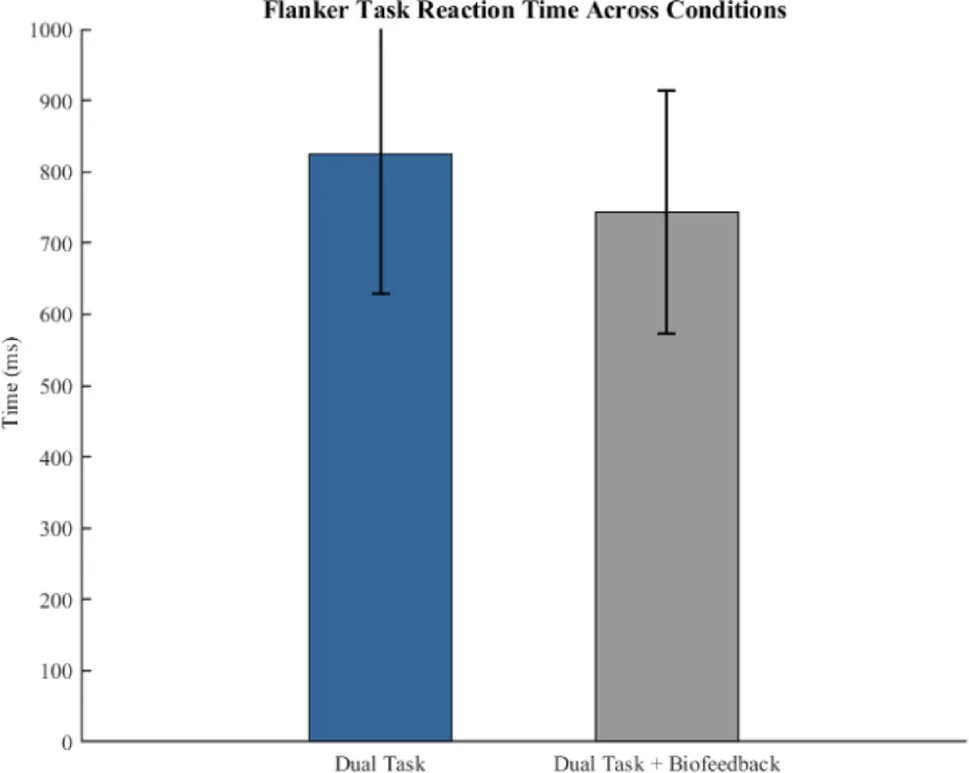
Flanker task reaction time across dual task and dual task + biofeedback condition

The flanker task was performed by the participants in dual task and dual task + biofeedback conditions. Figure 3 shows the performance in the flanker task in these two conditions. While there was a small difference (an average of 88% accuracy in dual task and 86% in dual task + biofeedback conditions), a paired t-test between the two conditions did not reveal a significant difference (*p =* 0.15). However, the average response time for button presses during the flanker task decreased significantly (*p* = 0.005) in the dual task + biofeedback condition compared to the dual task condition (825 ms vs. 744 ms, Fig. 4).

### Brain activity

**Fig. 5:**
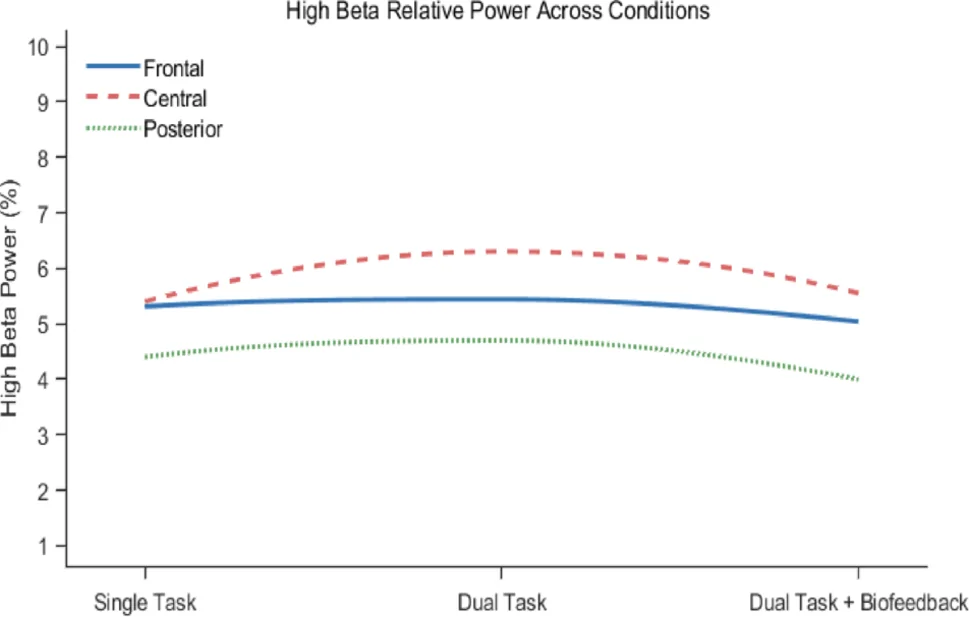
Relative power for high beta across different conditions for frontal, central and posterior regions of the brain

Figure 5 shows the mean values of high beta relative power across 3 tested conditions in frontal, central and posterior brain regions. The repeated-measures MANOVA revealed a statistically significant multivariate effect of Condition on high beta relative power (F(6, 64) = 2.686, *p* = 0.022, partial η^2^ = 0.201). Follow-up univariate tests (ANOVA) using a Greenhouse-Geisser correction were used to identify in which brain regions the significant differences occurred. These tests indicated a significant effect of condition on brain activity of the central part of the brain (F(1.89, 32.14) = 4.193, *p* = 0.026, partial η^2^ = 0.198). Post-hoc pairwise comparisons were then conducted across the 3 tested conditions for the central region of the brain. A significant increase in relatively high beta power in the central region of the brain was observed in the dual-task condition, compared to the single-task condition (*p =* 0.015). When compared with the dual task + biofeedback condition, the relatively high beta power was significantly reduced from the dual task condition (*p* = 0.023). Post hoc comparisons with the Benjamini-Hochberg Procedure confirmed these significant pairwise differences.

**Fig. 6:**
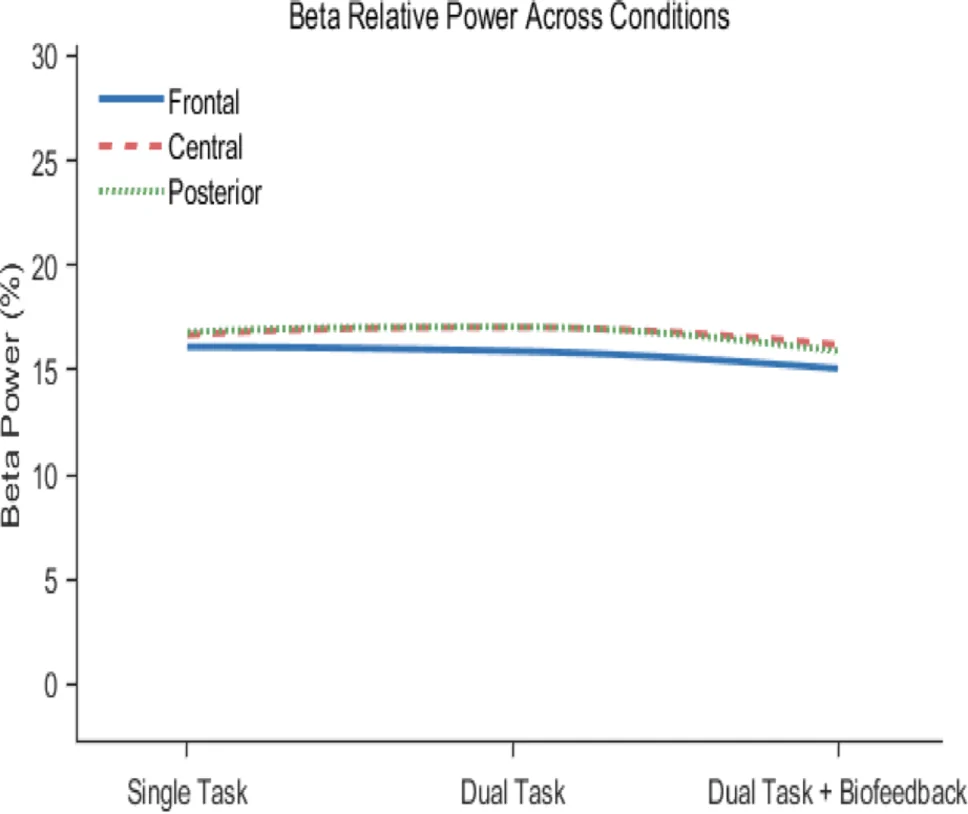
Relative power for high beta across different conditions for frontal, central and posterior regions of the brain

For relative power beta (Fig. 6), MANOVA did not reveal any global significant differences (F(6,66) = 1.57, *p* = 0.168, partial η^2^ = 0.188), so further statistical analysis was not conducted.

## Discussion

To the knowledge of the authors, this is the first study investigating how body movement and brain activities change in conditions of (1) single task, (2) cognitive-motor dual task, and (3) dual task with auditory pacing biofeedback assisting the movements. One main outcome of this study is that dual tasks significantly reduced ROM and increased COV of joint angle for both the left and right ankle joints during dual-tasking, suggesting some impairment in movement. This is further supported by the velocity results, particularly for the right ankle, where velocity decreased significantly in the dual-task condition. This aligns well with previous studies, which reported that the ability to perform foot taps was reduced by additional cognitive load (Manicioppi et al. 2022, Park and Brünken 2014). Furthermore, the biomechanical findings also align with previous gait studies, which reported that dual tasks significantly reduced gait velocity (Yogev-Seligmann et al. 2010; Hollman et al. 2007) and ankle dorsiflexion (Tavakoli et al. 2016) but significantly increased variability in ankle joint (Wang et al. 2021) and tibialis anterior and soleus muscle coactivation (Hallal et al. 2013) during walking.

Such changes can be explained by the greater processing effort required to respond to complex visual stimuli, resulting in a delayed motor response (Bauer 2006). This was supported by EEG data in this study, which showed significant increases in relative high beta power across the entire brain region in dual-task condition compared to the single-task condition (Please see Figs. 5 and 6). This highlights the increased cognitive stress during a dual-task (Baker 2007), which is expected given the nature of the flanker task (an attention-based task). Dual tasks cause interference, which can be described using the bottleneck theory. This theory states that processing a response to one task is interrupted as long as the other task is carried out in the central stage (Pashler 1994). In our study, we came across some inconsistencies between left and right ankle joint velocities. This could be attributed to lateral dominance and a bias toward the dominant foot, which was also observed in a similar study (Fearing et al. 2001). Another study (Numata et al. 2025) highlighted lower variability in alternating foot tapping than in symmetrical foot tapping, suggesting additional cognitive load on lateral dominance. Relative high beta power results reported in this study have been consistent with literature in these two conditions. This is also a good measure of cognitive load, as a reading of relative power two or more standard deviations above the mean can be considered excessive beta activity (Clarke et al. 2001).

It was interesting to note that additional auditory pacing biofeedback during the dual task (the dual task + biofeedback condition) returned the mean values of certain kinematic parameters (Left and Right ankle ROM, right ankle velocity) in a similar range to the single-task condition, and there were no statistically significant differences from the values found in the ST condition. Previous studies (Wittwer et al. 2013) observed improvements in gait with metronome feedback. This study found that auditory pacing biofeedback could still improve body movement when people performed an additional mental task concurrently, and that the performance of the mental task was not degraded by biofeedback.

The results showing improved body movements in the dual-task + biofeedback condition can be explained by changes in high beta activity of the brain. While significantly raised in the dual-task condition, particularly around the central region, the high beta relative power in the central area of the brain decreased significantly in the dual task + biofeedback condition, returning the values closer to the single task condition. Matching the foot taps to the beat of the metronome, may incorporate inhibitory processing, introducing some level of cognitive load (Park & Brunken 2014). However, the significant reduction in high beta power observed in this study suggested lower mental stress, anxiety, and arousal (Pino and Romano 2022). Participants may have felt less cognitively challenged by the auditory pacing biofeedback, as reduced beta activity correlates with decreased sensory processing and attentional demands (Herath 2001). The lower stress might also explain the surprisingly better results from the mental flanker task. In addition to the improved foot tapping performance, the auditory pacing biofeedback that assisted tapping performance also improved the mental flanker task results as participants had significant decreases in response time, while maintaining the degree of accuracy. The significant changes in high Beta (24–39 Hz), rather than beta (13–24 Hz) frequency band, align well with previous studies showing that high-beta activity is more closely associated with attentional control (Palacios-García et al. 2021) and cognitive–motor interference (Nougaret et al. 2024), making it more sensitive to dual-task demands and externally driven pacing than lower-beta activity.

While many biofeedback devices provide real-time feedback regarding movement error, some studies have classified metronome signals as a type of biofeedback, even though the cue itself acts primarily as pacing rather than error corrections (Gouda and Andrysek 2024). The metronome-type biofeedback can contribute to entrainment effects, as there might be synchronization of brain waves with the auditory metronome beats. This study purely focuses on the kinetic adaptations of the ankle joint rather than temporal precision. There was no single-task + biofeedback, which potentially helped identify any entrainment effects. There is a possibility that the changes in ankle motion data and EEG signals in the dual task + biofeedback condition were contributed to by the entrainment effects, as there might be synchronization of brain waves with the auditory metronome beats. Auditory-motor interactions are well documented as potential contributors to entrapment (Bispham 2006). While decreased beta power is consistent with decreased cognitive load during dual tasks (Foldal et al. 2022), which may be associated with the observed motor improvement in DT+ condition, it is to be acknowledged that these oscillatory changes can also be explained by entrainment effects (Bispham 2006). Meanwhile, beta oscillations are also known to play a key role in sensorimotor integration, maintenance of the current motor set, and motor system stabilization (Engel and Fries 2010; Baker 2007; Spitzer and Haegens 2017). From this perspective, the changes in high beta activity during DT and DT+ conditions may also reflect the motor system’s effort to preserve stability under increased control demands. When rhythmic motor behaviour is externally paced, reliance on internally generated timing and interoceptive monitoring is reduced, shifting control toward exogenous mechanisms and lowering the demands on top down control networks (Dohata et al. 2025).

Dual tasks have been widely researched to understand cognitive processing and cognitive interaction (Monteor-Odasso et al. 2017; Hunter et al. 2018). Meanwhile, the results of this study on the benefits of auditory pacing biofeedback for movement performance among people distracted by a secondary mental task can inform potential applications. For example, they could potentially support the design of devices that provide feedback to remind drivers of mental distraction and people who have lower cognitive ability of potential tripping hazards, giving the notion that appropriate biofeedback may improve body movement in cognitively challenged conditions. Biofeedback can be visual, audio, and vibrotactile, and it can give users different forms of instructions (e.g., simple beep sound vs. voice instructions). Another interesting application where combining biofeedback with a dual task could be beneficial is to understand cognitive capabilities. The experimental design utilized within this study can provide a gateway to understand whether populations with active cognitive decline or mild dementia can respond and perform motor tasks positively due to the addition of biofeedback.

There are, however, some limitations to this study. One notable point is that this study recruited more female participants, although the gender imbalance likely did not affect the findings. This is because previous studies have confirmed that differences in multitasking capabilities largely depend on the type of multitasking rather than gender (Lui et al. 2020). Furthermore, another study showed no significant difference in multitasking/dual tasking cost and other measures (processing speed, reaction time, accuracy, etc.) between genders (Hirsch et al. 2019). In terms of the motor task utilized within this experimental design, while participants tapped at natural and comfortable paces in the single-task condition, the double heel and toe taps could make the movement task very specific, and it may not be possible to generalize the findings to other movement tasks. Further limitations include the absence of the analysis of time-based analysis such as inter-tap intervals, their variability or synchronization error.

Meanwhile, there is a lack of significant difference (88% vs. 86%, *p* = 0.15) within the flanker task accuracy, which may reflect a ceiling effect. The relatively long stimulus presentation time (1.5s) and inter-stimulus interval (1s) may have been insufficient to tax cognitive resources. As such, it cannot be ruled out that the flanker task performance was masked by the ceiling effect. Future studies can increase task difficulty, for example, by shortening stimulus presentation time (300–500 ms), separating the analysis of incongruent and congruent trials.

Unlike gait, which consists of multiple segments of complex movement, foot tapping provides a much simpler approach for kinematic analysis. Although the ankle joint is a synovial hinged joint with 6 DOF (Degrees of Freedom) (Brockett and Chapman 2016), for this study, only the plantar flexion and dorsiflexion on the sagittal plane were observed during the foot tapping process. The range of motion, as explained in Sect.  2.4.1, was calculated by finding the average difference between the plantar and dorsiflexion of the ankles, and the velocities were calculated by utilizing the peaks and troughs found in Xsens data, which was a result of the motion of the ankle joint during foot tapping. Future work may include investigating different modes of dual-tasking that target different aspects of cognitive processing capabilities (e.g., Mental calculation or other attention-based tasks). Other modes of biofeedback, such as auditory feedback from the metronome, can also be investigated. This may include visual feedback or vibration-based feedback. The effect of different modes of biofeedback on one’s cognitive ability can provide new insights that could be useful for understanding a person’s cognitive ability and information processing capabilities. Furthermore, different modes of biofeedback could be paired with other, more complex or more natural motor tasks such as TUG (Timed Up and Go) or walking. This may reveal further interesting insights into how movement changes can reflect cognitive state.

## Conclusion

While mental-physical dual tasks worsened physical performance in foot tapping, performance in both tasks improved when biofeedback assisted foot tapping was added. An increase in high-beta activity across the brain during the dual-task condition, compared to the single-task condition, highlights the imposed cognitive strain and helps explain the worsened foot-tapping performance in the dual-task condition. When beats were provided from a metronome, reminding participants of their normal tapping frequency, there were significant decreases in high beta activity in relative power, accompanied by improved performance in both mental and physical tasks. The reductions in beta activity could be a result of reduced cognitive load. It could also be caused by entrainment effects or adaptive cognitive–motor resource reallocation, which were not investigated in this study. Further research should investigate the effects of other dual tasks (working memory, pattern recognition, etc.) and different methods of biofeedback. Furthermore, it can be investigated whether this protocol can be utilized to detect or diagnose potential cognitive impairment, or even dementia.

## Data Availability

The data supporting this study’s findings are available from the corresponding author upon reasonable request. No datasets were generated or analyzed during the current study.
